# Employing a spatio-temporal contingency table for the analysis of cork oak cover change in the Sa Serra region of Sardinia

**DOI:** 10.1038/s41598-018-35319-1

**Published:** 2018-11-16

**Authors:** Sandro Dettori, Maria Rosaria Filigheddu, Giovanni Deplano, Juan Escamilla Molgora, Maddalena Ruiu, Luigi Sedda

**Affiliations:** 1 Department of Agricultural Sciences, University of Sassari, Viale Italia 39, 07100 Sassari, Italy; 2 Lancaster Environmental Centre, Lancaster University, Lancaster, LA1 4YQ United Kingdom; 3 Centre for Health Informatics Computing and Statistics (CHICAS), Lancaster University, Lancaster, LA1 4YQ United Kingdom

## Abstract

Land cover change analyses are common and, especially in the absence of explanatory variables, they are mainly carried out by employing qualitative methods such as transition matrices or raster operations. These methods do not provide any estimation of the statistical significance of the changes, or the uncertainty of the model and data, and are usually limited in supporting explicit biological/ecological interpretation of the processes determining the changes. Here we show how the original nearest-neighbour contingency table, proposed by Dixon to evaluate spatial segregation, has been extended to the temporal domain to map the intensity, statistical significance and uncertainty of land cover changes. This index was then employed to quantify the changes in cork oak forest cover between 1998 and 2016 in the Sa Serra region of Sardinia (Italy). The method showed that most statistically significant cork oak losses were concentrated in the centre of Sa Serra and characterised by high intensity. A spatial binomial-logit generalised linear model estimated the probability of changes occurring in the area but not the type of change. We show how the spatio-temporal Dixon’s index can be an attractive alternative to other land cover change analysis methods, since it provides a robust statistical framework and facilitates direct biological/ecological interpretation.

## Introduction

Cork oak is an evergreen tree species whose distribution spans from the Iberian Peninsula (Portugal, Spain) and North Africa (Morocco, Algeria, Tunisia) to Corsica (France) and Italy, covering 2.1 million hectares^1^. In Italy, cork oak woodlands are concentrated in Sardinia, where they cover 137941 ha (2010 census)^2^, with an additional 489877 ha (equivalent to 20% of the whole Sardinian territory) of mixed cork oak systems in which cork oak coexist with other tree species or within agricultural systems. The widespread presence of cork oak in the territories surrounding the Mediterranean basin is supported by the high economic, cultural, and ecosystemic (biodiversity, tourism etc.)^3^ value of the cork for rural and peri-urban communities^4–6^. Cork oak forest regeneration, however, is facing difficulties due to the presence of livestock activities and demographic changes^7–9^, or due to competition with other tree species when the cork oak forests fall into disuse owing to the contraction of the cork economic market^10^. Therefore, despite the European trend of forest expansion^11,12^, Mediterranean cork oak forests are experiencing large scale shrinkage^13^. This is due to the regeneration problems described above and the increases in mean annual temperatures, tree diseases, overgrazing and environment degradation^14^, which often overwhelm cork oak afforestation efforts^13^. It is estimated that almost half of all cork oak forests have been lost over the last 100 years^14^.

The cork oak forests of Sa Serra in Sardinia are considered historical forests of high heritage value. Grazia Deledda (Nobel Prize for Literature in 1926), in one of her short novels written at the beginning of 1900, described the cork oak forests of Sa Serra as being “of ancient history”^15^.

In this work, we estimated and mapped the local rate of cork oak forest cover change in the Sa Serra region of Sardinia. The null hypothesis is that within the study region there were no changes in cork oak forest cover in the last 18 years (1998–2016). Hypothesis testing was performed using a method belonging to the family of marked point processes^16^, which are point patterns representing the distribution of categorical labels (in this case, land cover) or continuous measurements^17^. Despite the large number of tests for marked point processes, few can handle bivariate or multi-variate interactions. For this reason, we decided to adapt Dixon’s index of segregation^18^, which is based on a nearest neighbour contingency table, on both spatial and temporal domains, in order to identify and quantify cork oak forest cover changes. Segregation is a measure of the interaction between two classes and can be defined as ‘the spatial pattern in which points from the same class are closer to each other’^19^, while association is the spatial pattern ‘in which points from different classes are closer to each other’^19^. In other words, two classes are segregated if the points of each class occur near individuals of the same class more frequently than random expectations^20^. Therefore, the null hypothesis stated above can be reformulated as: *within the study region there is no association nor segregation in cork oak forest cover changes between the two surveys (1998 and 2016)*. This is equivalent to stating that any changes are those expected as a result of random labelling^18^.

The literature is rich in studies on land use and land cover changes, and their identification and delineation^21,22^. These studies can be classified into two groups: those that analyse the intersection of the results of two land cover classifications^23,24^ and those that employ (often hierarchical) models (i.e. logistic regression)^25^. The latter are preferred when explanatory variables and more than two surveys are available (see^26,27^ for a comparison of different spatially explicit models/learning machines), although changes in labels, when dealing with more than two classes, can raise challenges in the modelling approach^28,29^.

When modelling land cover changes in the absence of explanatory variables and when explicit biological/ecological interpretation of the processes determining the changes is required, the use of statistical indices^30–35^ is preferred. There are several indices based on nearest neighbour or autocorrelation^36^. The advantages of using Dixon’s index of segregation can be summarised as follows: it accounts for interactions between two classes^37^; provides one-class-versus-rest-of-the-classes tests, and class-specific tests; and it is robust in detecting interactions over small distances^19^. Finally, Dixon’s index of segregation is easy to interpret and allows the identification of segregation, association or none of the above based on a pre-defined significance level^38^.

In this paper, we first modified Dixon’s index of segregation in order to account for the temporal dimension and correct for edge effects. In addition, we have implemented the index in a neighbourhood moving framework that allows for the calculation of the Dixon’s index of segregation at each point of the study area (local statistic) instead of producing a single value for the entire area (global statistic). This modified Dixon’s index of segregation was applied to the changes in cork oak forest cover in the Sa Serra region of Sardinia. We then produced a map of Dixon’s index of segregation in order to identify areas with positive (segregation– cork oak forest cover extension) or negative (association– cork oak forest cover erosion) Dixon’s values. As we stated above, this method allows us to map the rate of change, because the Dixon’s index of segregation is equivalent to a log odds ratio, that is, the odds that the change of one land cover to another occurs based on the proportion of each land cover in the neighbourhood. In addition to the Dixon’s index of segregation, we applied a spatially explicit binomial-logit generalised linear model to the cork oak forest cover changes in order to obtain for each point in the grid the probability of presence of cork oak forest cover given the presence/absence in the two surveys. The spatial binomial-logit generalised linear model results are then compared with the segregation map and differences are discussed. Finally, we modelled the grid point Dixon’s values by employing a generalised geostatistical linear model in order to determine the effect of other covers on cork oak forest cover changes.

## Materials and Methods

### Study area

**Figure 1:**
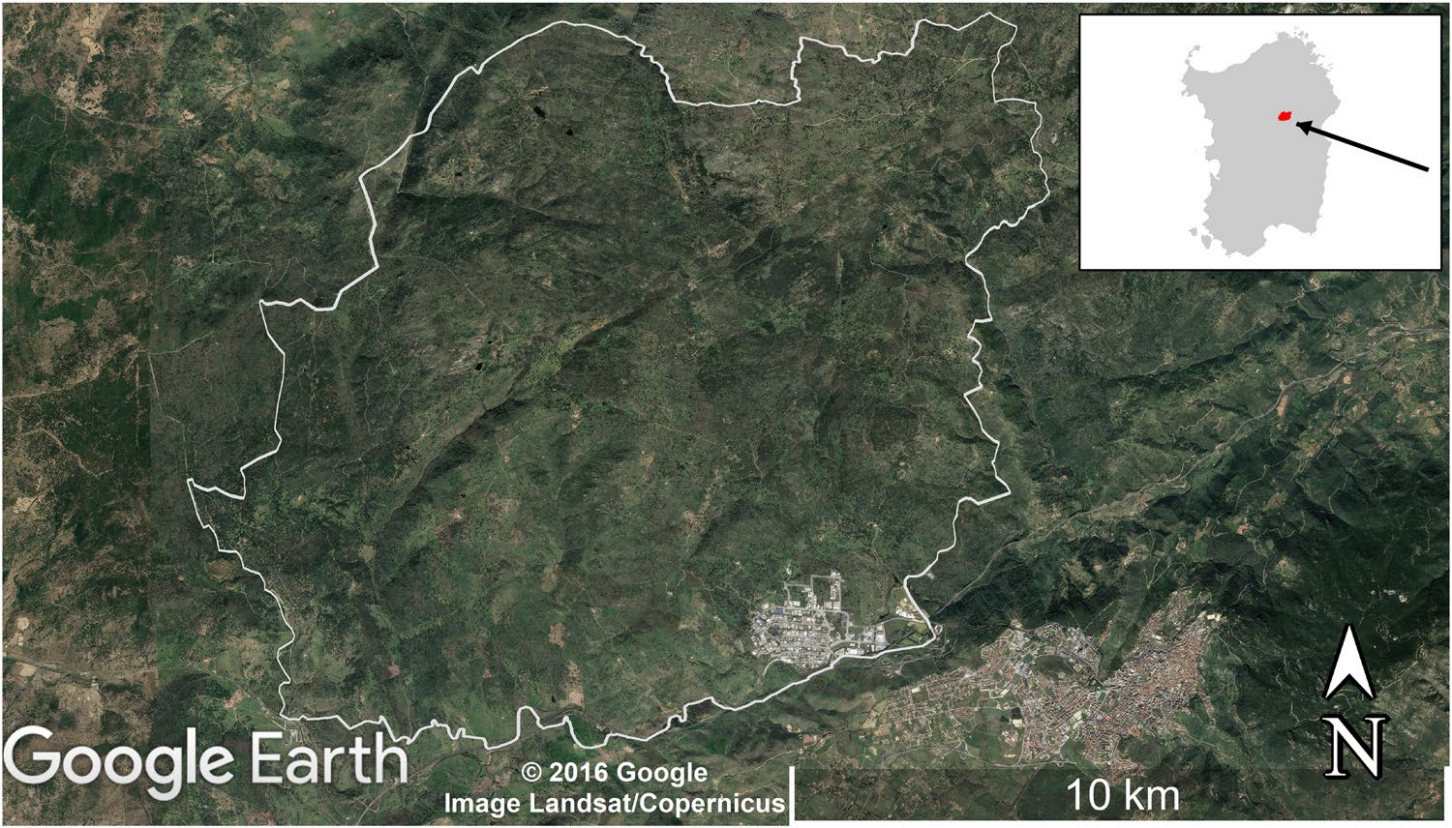
Study area of Sa Serra (within white contour). Background from Google Earth (Image: Google, Landsat/Copernicus). Source administrative limits: http://dati.regione.sardegna.it/dataset/limiti-amministrativi-comunali under CC-BY-4.0 licence.

The study was carried out in Sa Serra (an area of 101 km^2^, spanning from 9°15’E and 40°40’N to 9°30’E and 40°30’N), a historic cork oak forest landscape on the island of Sardinia (Italy) (Fig. 1). The area encompasses four municipalities: Nuoro, Orune, Orani and Oniferi. Sa Serra elevation extends from 350 m to 830 m a.s.l., and the climate conditions are suitable for cork oak regeneration and growth^39^.

### Land cover data

The 1998 land cover map for Sa Serra was obtained from Sedda and colleagues^2^. This was derived from an unsupervised classification of 1998 orthorectified aerial photographs at 1.0 m resolution, which allowed the delineation of the area into polygons that are homogenous in colour, smoothness, compactness and shape. Subsequent photo-interpretation of each polygon followed by field validation of 20% of them, allowed the identification of 5 land cover categories: agricultural (croplands, arable land, orchards, vineyards and related infrastructures and buildings), cork oak forest (forest with more than 25% of cover by cork oak tree canopy in a minimum area of 5000 m^2^), other forest (mixed forest and mature shrub-lands with less than 25% of cover by cork oak tree canopy), natural pasture (meadows, unproductive grass and shrubs), and urban (urban buildings and lands, transport network). The definition of ‘other forest’ follows the FRA-FAO (Forest Resource Assessment Programme, Food and Agriculture Organization of United Nations^40^) guidelines: i.e. that it has a minimum area of 5000 m^2^ and a tree canopy cover of more than 10% of the area. The forest density was estimated using a grid of points overlaid on the photographs where each grid represents a 70 m by 70 m square containing one point per m^2^. The tree canopy density is then evaluated by simply counting the number of points on the grid that are contained within the tree canopy. If at least 500 points (corresponding to the FRA-FAO 10% figure) are contained within the tree canopy, then the grid contains forest; if at least 1250 points (25%) are contained within cork oak tree canopy, then the grid contains cork oak forest (full details of the 1998 land cover classification can be found in Sedda *et al*.^2^).

The 2016 land cover map for the same area was produced by editing the land cover maps from 1998 using colour aerial photographic images (orthophotos) of Sardinia taken in March 2016 at a nominal ground resolution of 0.2 m, provided by AGenzia per le Erogazioni in Agricultura (AGEA)^41^. The editing (merging, splitting and re-classifying polygons using ArcMap software^42^) was carried out during the photointerpretation and field validation (this time on 40% of the polygons) phases. Canopy density is again calculated as described above.

**Figure 2:**
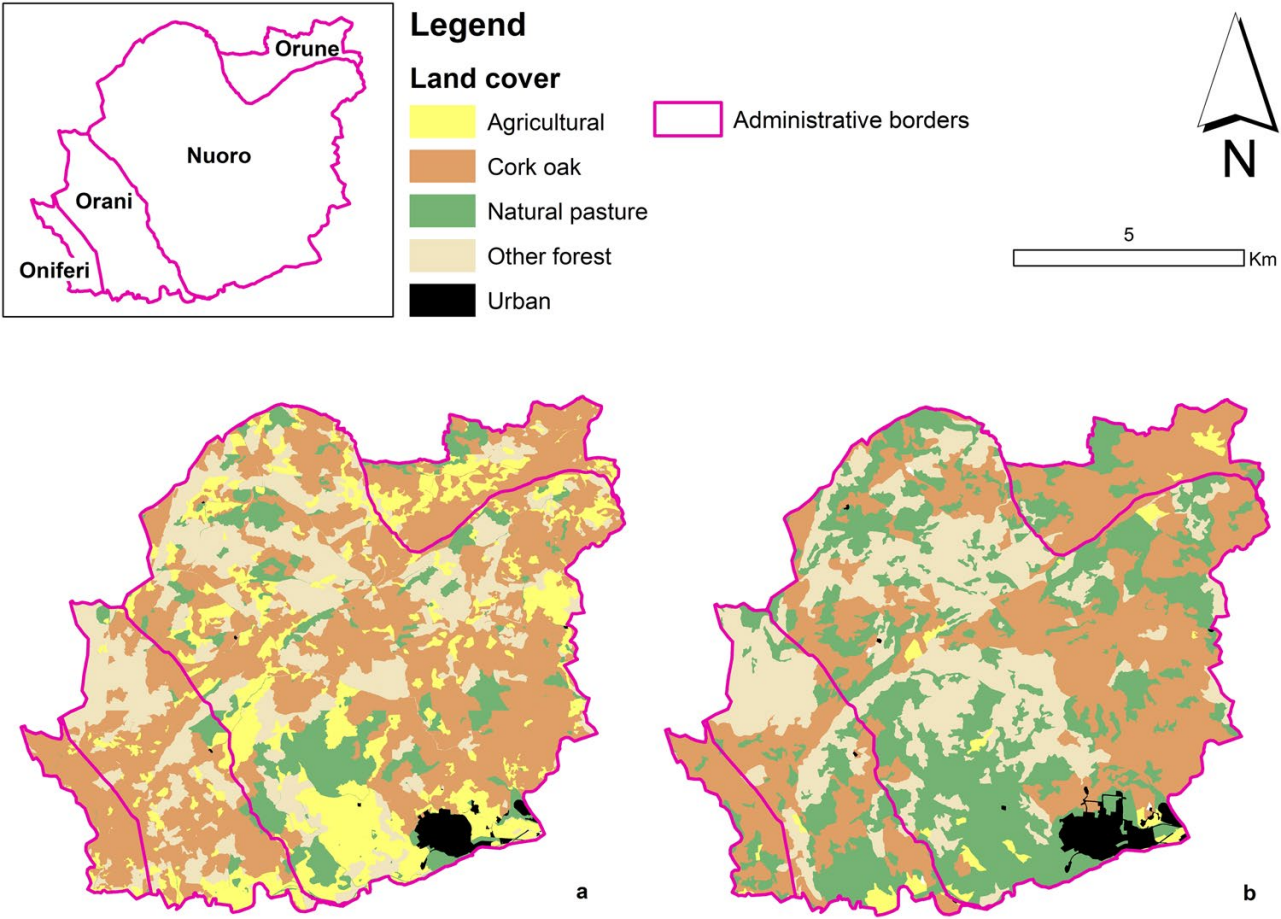
Land cover in the Sa Serra region in 1998 (**a**) and 2016 (**b**). Maps were made using ArcMap 10.4 (http://desktop.arcgis.com/en/arcmap/). Source administrative limits: http://dati.regione.sardegna.it/dataset/limiti-amministrativi-comunali under CC-BY-4.0 licence.

It is important to note that the photointerpretation phase in both 1998 and 2016 was carried out by local scientists that are familiar with the land use and land cover of the area. The two maps are shown in Fig. 2. Each land cover polygonal map (1998 and 2016) was converted into raster format at a resolution of 50 m, in order to detect the smallest forest units (whose minimum size is 5000 m^2^) and at the same time reduce the number of points affected by orthorectification and classification errors. In each raster, the total number of grid nodes is 40548.

### Statistical analysis

#### Spatiotemporal Dixon’s index of segregation

Dixon^18^ proposed a nearest neighbour (spatial) contingency table for the analysis of segregation of populations or species, based on the frequency of the different groups of individuals (or species), the label of the nearest neighbour, and the geometric configuration of the points. This statistic is valid when the locations are sparse or in a regular grid (completely mapped data). The seminal work of Dixon^18^ considered a spatial co-occurrence scenario only (i.e. it excludes the temporal domain). In this work, we modified Dixon’s index of segregation to allow the following adjustments (notation as in^18,37^):

- To be applied on both temporal and spatial dimensions simultaneously,
- Local instead of global metric: the 2 by 2 contingency table (Table 1) is constructed within a square spatial window of size *d* around each point of the grid (the original proposition of Dixon is a global statistic with a global 2 by 2 contingency table),

**Table 1:** The 2 × 2 contingency table applied to each neighbourhood of size *d*.

Label in 1998	Label in 2016		Total
	Cork oak forest	Other	
Cork oak forest	*Naa*	*Nab*	*Na*
Other	*Nba*	*Nbb*	*Nb*
			*N*

Within the neighbourhood, *Naa* is the number of points classified as ‘cork oak forest’ in 1996 that remain unchanged; *Nab* is the number of points that were labelled ‘cork oak forest’ in 1996 but are not in 2016; *Nba* is the number of points labelled ‘other’ in 1996 and ‘cork oak forest’ in 2016; *Nbb* is the number of points classified ‘other’ in 1996 that remain unchanged. *N* is the sum of points in 1998 and 2016 (Na+Nb) within the spatial window of size *d*.
- Window size, *d*, is optimised based on the general statistical significance of the local index (see below),
- Edge correction: we applied a weighted edge correction of the index^43^ by locally enlarging *d* until a sufficient number of points are included (i.e. to satisfy the condition that *Naa* and *Nab*, as in Table 1, are larger than 4; which constrains to positive values the probability that a quartet of points are cork oak forest or other forest; equations for these probabilities are provided by Dixon^18^),
- Point parameters: the number of points with shared nearest neighbour (parameter *Q* in^18^) ranges from 2 to a maximum of 5 (4 corresponding to each side of the grid cell at time *t*, plus 1 collocated at time *t* + *1*).

The extension to the temporal domain is obtained by defining the nearest neighbour of a location *p* at time *t* (a point in the 1998 land cover), as the co-located point *p* at time *t* + *1* (in the 2016 land cover). In addition, in order to preserve the assumption of random labelling for the null model, we restricted the comparison to being solely between cork oak forest cover and other covers, where ‘other covers’ includes agricultural, natural pasture, other forest and urban covers. In fact, in the presence of three or more classes, null model selection is confounded by the existence of multiple processes (i.e. co-occurrence of strong and weak interactions that make the statistic uninterpretable), including processes that affect the entire joint distribution of the classes (for example, a strong environmental gradient)^44^. Therefore, converting the multi-class case into a bivariate one for spatial segregation comparison removes the risk of having large degrees of freedom in the presence of confounders, and allows a more accurate approximation of the joint population. The analysis was thus performed on binary variables (one for each year) such that 1 denoted cork oak forest cover and 0 any other land cover. The major drawback of the proposed methodology is then that the calculated index of segregation cannot tell us anything about the bivariate class-specific (and locally significant) association or segregation (i.e. cork oak forest versus other forest, or other forest versus agriculture etc…). For this reason, we have created a geostatistical additive linear model of the Dixon’s index values to ascertain the relationship between segregation/association in cork oak forest cover with presence/absence of other covers.

The measure of Dixon’s index of segregation, *Saa*, for the cork oak forest cover at a given local neighbourhood *d* is obtained from the elements of the contingency table described above (Table 1) and calculated as:

1 $$Saa=\,\mathrm{log}(\frac{Naa/Nab}{(Na-1)/Nb})$$

where *Na* is the sum of number of points classified ‘cork oak forest’ in 1998 and 2016, and *Nb* is the sum of number of points classified as ‘other’ in 1998 and 2016 (within the window *d*). Testing this index against the null model of random labelling requires a ${{\chi }^{2}}_{2}$segregation statistic, *C*, based on the expectations, variances and covariances under random labelling^18^:

2 $$C=\frac{{z}_{aa}^{2}+{z}_{bb}^{2}-2r{z}_{aa}{z}_{bb}}{1-{r}^{2}}$$

where *r* is the correlation between *Naa* and *Nbb* (the subscripts *aa* and *bb* indicate unchanged cork oak forest cover and unchanged other cover respectively, as described in Table 1). The calculation of *C* requires two standard *z* score statistics: *z*_*aa*_ and *z*_*bb*_:

3 $${z}_{aa}=\frac{Naa-{\mathbb{E}}(Naa)}{\sqrt{Var(Naa)}}$$

4 $${z}_{bb}=\frac{Nbb-{\mathbb{E}}(Nbb)}{\sqrt{Var(Nbb)}}$$

where ${\mathbb{E}}$*(Naa)* and ${\mathbb{E}}$*(Nbb)* are the expected number of points classified ‘cork oak forest’ and ‘other’ respectively that remain unchanged from 1998 to 2016 under random labelling. We notice from equations (3) and (4) that the *z*-scores do not depend on the interaction between the two land cover as they do in the *Saa* equation (1) (for this reason *Saa* is also known as the pairwise segregation index) and are often referred to as a species/neighbour specific test^38^. *z*_*aa*_ and *z*_*bb*_ also have useful ecological interpretations; indicating clustering of the land cover for values significantly larger than 0 (above 1.96 at a 95% confidence level), or sparsity for values significantly lower than zero (below -1.96 at a 95% confidence level).

The *p*-values for *z*_*aa*_ and *z*_*bb*_ are obtained from an asymptotic normal distribution approximated by a Monte Carlo randomization (using 5000 simulations^37^). The *p*-value for *C* is obtained from the same Monte Carlo randomization, which approximates the asymptotic ${{\chi }^{2}}_{2}$ distribution. The segregation analysis was performed using a significance level of 5%^45^.

As recommended by Dixon^18^, if *C* is significant then the overall test of segregation *Saa* can be interpreted by looking at its value. If *Saa* is close to 0, the cover changes are as expected under random labelling. If *Saa* is larger than 0, the number of new cork oak forest cover points is larger than its expected value under random labelling (cork oak cover intensification/expansion). Finally, if *Saa* is lower than 0, the number of remaining cork oak forest cover points is lower than its expected value under random labelling (cork oak forest cover reduction/erosion process).

As described at the beginning of this section, Dixon’s index of segregation is applied at each grid point within a window *d* moving across all the points of the study area. The choice of the neighbourhood dimension *d* is therefore important. In fact, the association between covers in the two time periods is dependent on the size and shape of the neighbourhood window^46,47^. The appropriate quadrat size for detecting segregation/association between the two land cover (window *d*) is chosen as the one that is associated with the largest number of significant points (i.e. points with Monte Carlo *p*-value for *C* smaller than 0.05). Specifically, we have repeated the analysis for different *d*: 100 m, 250 m, 500 m, 1000 m and 1500 m, and selected the *d* associated with the largest number of points having a statistically significant Dixon’s index value.

The map of the *Saa* values is produced to visualize the degree to which the cork oak forest cover erosion or expansion is taking place in the area of Sa Serra. The uncertainty at each point is estimated as the standardised 95% interquartile credible interval (as obtained from the Monte Carlo randomizations) at its respective point-estimated *Saa*.

#### Spatially explicit binomial-logit generalised linear model

The Dixon’s index of segregation was compared with a spatial binomial-logit generalized linear model^48^. One of the first limitations in modelling land cover/use changes with a binomial model is in the definition of the response variable. The simplest approach is to consider the changed/unchanged classes. However, this approach considers only the ‘changed’ information and does not distinguish between change from cork oak forest cover to other cover or from other cover to cork oak forest. For this reason, logistic regression models are usually applied individually to each land cover class and only consider whether the class changed or not (an example is provided in Peppler^25^). While the dynamics are not analysed, this approach allows the inclusion of an explicit autocorrelation model (i.e. a variogram^16^) and explanatory variables, which can describe the trend in changes. The latter is the main advantage of using a model rather than a point pattern statistic such as Dixon’s index of segregation.

We modelled the presence/absence of cork oak forest in 2016 conditioned to the presence/absence of this class in 1998. The full statistical formulation of the model is as follows:

5 $$Y(x)|S(x) \sim Bin(2,P(x))$$

6 $$logit\{P(x)\}=\alpha +S(x)$$

7 $$S(x) \sim GP(0,{\sigma }^{2}\rho (u))$$

8 $$\rho (u)=\exp (-u/\varphi )$$

where *Y(x)* is the presence of cork oak forest at an arbitrary location *x*; *S(x)* is the spatial Gaussian process with process mean 0, variance *σ*^2^ and (positive definite) spatial autocorrelation function *ρ(u)*, where *u* is the Euclidian distance between points, and $\varphi$ the spatial range. *Y(x)* follows a Binomial distribution with 2 trials (1998 and 2016) and probability of successful outcome (cork oak forest presence) *P(x)*, where *P(x)* depends on the average frequency of cork oak forest in the area, *α*.

We fixed the values for the correlation function: $\varphi$=5000 m and *σ*^2^ = 1. All the other parameters are estimated by model fitting with a Langevin-Hastings MCMC algorithm^49^.

A full description of the model can be found in^48^. Calculations are performed using the binom.krige function from the R^50^ package ‘geoRglm’^51^.

#### A geostatistical additive model for Dixon’s index segregation values

Finally, we modelled the significant *Saa* values, i.e. the Dixon’s index of segregation values obtained from equation (1), at each location, *x*, using a linear geostatistical model of the form:

9 $$Saa|Z(x) \sim N(f(x),{\tau }^{2})$$

10 $$f(x)=\beta {\bf{X}}+{\bf{Z}}(x)$$

where *Saa* is normally distributed with mean *f(x)* and variance *τ*^2^. *f(·)* is the link function, $\beta$is a vector of coefficients for **X**; **Z** is a column vector of spatial normally distributed random effects (Gaussian stochastic process) with mean zero and spatially autocorrelated covariance function (which is equivalent to *S(x)* in equation (7)); and **X** is the matrix of independent variables (covariates)^52^. Parameterization is obtained via Maximum Likelihood Estimation (MLE) under Gaussian assumptions on the process and errors. The linear geostatistical model is fitted using the R^50^ package ‘geostatsp’^53^.

The model was estimated for **X** using the agricultural cover, other forest cover, and natural pasture cover variables in 2016.

## Results

### Spatiotemporal Dixon’s index of segregation

From the transition matrix of the two land cover maps, the observed cork oak forest loss was 8% (Table 2 and Fig. 2) due to loss of density of cork oak within other forest types or in natural pastoral systems. In fact, by regional law definition (see Discussion), cork oak forest must have cork oak canopy cover greater than 25% (http://dati.regione.sardegna.it/dataset/carta-delluso-del-suolo-in-scala-1-25-000-elementi-poligonali-2008); therefore, if this density decreases, the cork oak forest is automatically re-classified as another land cover. In addition, Table 2 shows a strong trend towards pastoral activities: between 7 and 10% of cork oak forests, other forest and agriculture were reclassified as natural pasture in 2016.

**Table 2:** Land cover changes from 1998 (rows) to 2016 (columns).

1998/2016	Cork oak forests	Other forest	Agricultural	Natural Pasture	Urban	Net
*Cork oak forest*	38	10	0	8	0	−8%
*Other forest*	5	26	0	7	0	+ 2%
*Agricultural*	4	2	2	10	0	−16%
*Natural pasture*	1	2	0	32	0	+ 22%
*Urban*	0	0	0	0	2	+ 0%

The net class change is reported in the last column. For example for cork oak forest this value is the result from the gains (5 + 4 + 1) reduced by the loss (10 + 8).

**Figure 3:**
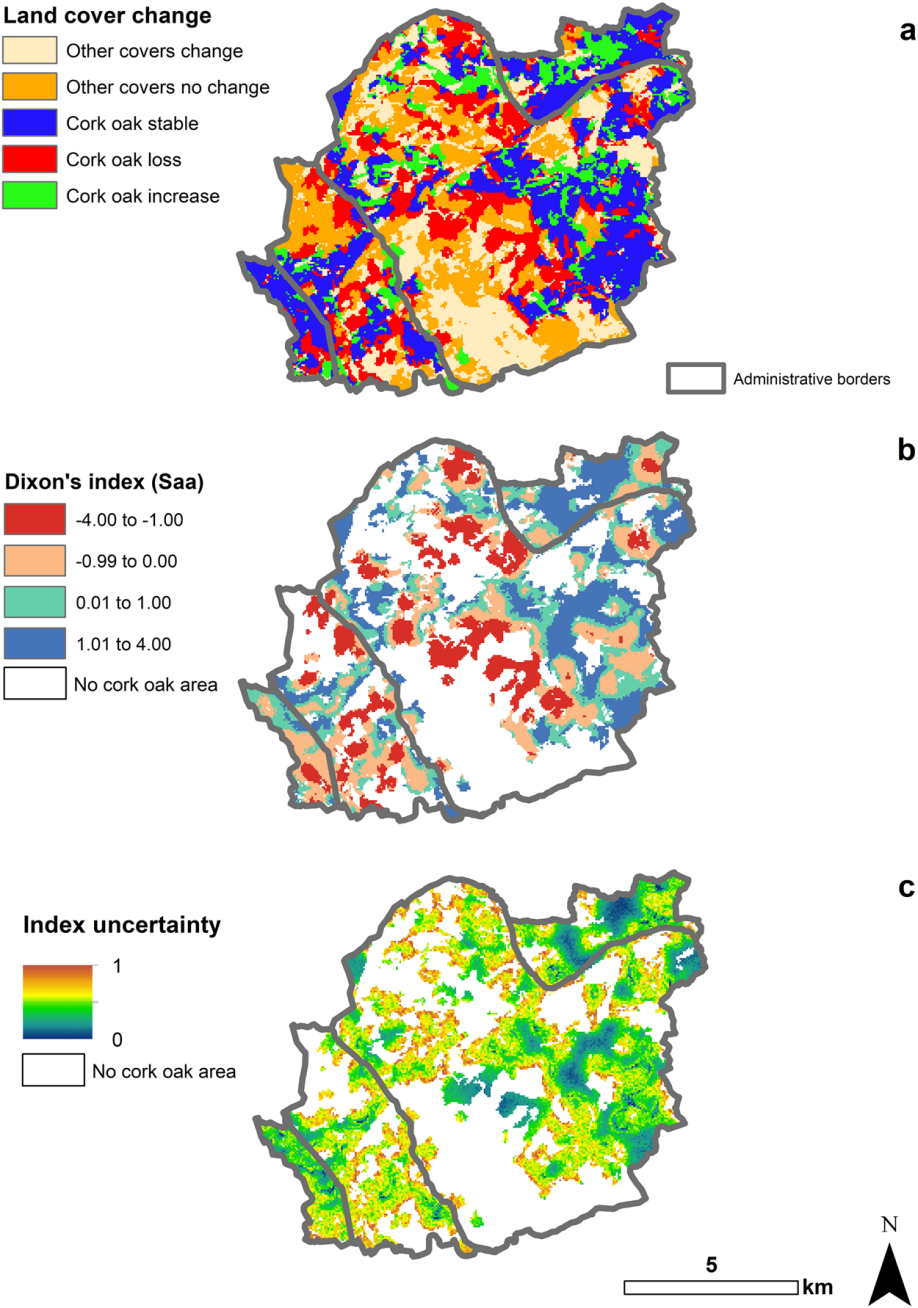
Land cover changes in Sa Serra during the period 1998–2016. Panel a: Land cover changes. Panel b: Dixon’s index of segregation. Panel c: Uncertainty of the Dixon’s index of segregation. The Dixon’s index of segregation (Panel b) has unlimited boundaries. Values larger than 0 means segregation, i.e. points with cork oak forest in 1998 are closer to points that became cork oak forest in 2016; conversely negative values mean association, i.e. points with cork oak forest in 1998 changed or are closer to point that changed from cork oak forest to another land cover in 2016. Larger is the value, larger is the rate of segregation, i.e. areas with values equal to −4 are experiencing a rate of change that is double from areas with values equal to -2. Standardised uncertainty varies from 0 to 1 (Panel c), where 1 means largest interquartile range (not possible to infer an accurate value of the index). Maps were made using ArcMap 10.4 (http://desktop.arcgis.com/en/arcmap/). Source administrative limits: http://dati.regione.sardegna.it/dataset/limiti-amministrativi-comunali under CC-BY-4.0 licence.

Table 2 shows the changes in land cover but does not say anything about the statistical significance of these changes or where these changes are taking place (a common issue in all the studies using global confusion matrices). By employing the spatio-temporal Dixon’s index of segregation, it was possible to quantify and map the intensity of cork oak forest cover changes and evaluate whether these changes were significantly different (at the 5% probability level) from a random labelling null model (Fig. 3).

Figure 3b,c are produced with a neighbourhood window (*d*) of 250 m, which is the window with the highest number of significant points (p-value < 0.05): 7175 compared to 7008 (100 m), 6217 (500 m), 1589 (1 km) and 239 (1.5 km).

All the four municipalities represented within Sa Serra had cork oak forest cover reduction; although this reduction is more intense in the centre and west-centre than the east-centre of the region (Fig. 3a). Figure 3b shows the map of the *Saa* as calculated in equation (1) at each point of the grid. The larger the value of *Saa*, the more extreme the association (positive or negative) between the two time periods. Negative values indicate that cork oak forest cover in 2016 is found less frequently than in 1998 (less frequently than expected under random labelling); while values larger than 0 mean that cork oak forest cover increased (more frequently than expected under random labelling thanks to the transformation of other covers into cork oak forest cover) between 1998 and 2016.

Finally, values of *Saa* close to 0 are consistent with no changes or changes that are expected under random labelling (expected random changes for a pre-defined level of significance).

Figure 3b statistically confirms the land cover transition map in Fig. 3a. The estimations are accurate (Fig. 3c) for the cork oak forest cover reduction in the centre of Sa Serra and cork oak forest expansion in East and North East of Sa Serra. Notable from Fig. 3c is that the most uncertain areas (in yellow towards red) are those that neighbour large areas without cork oak forest in both 1998 and 2016 (the white areas in Fig. 3b,c) and where other changes are also taking place (Fig. 3a). As expected, the largest positive values (cork oak forest cover intensification), are concentrated where there is a combination of transformation of other covers into cork oak forest cover and preservation of the existing cork oak forest cover (blue colours in Fig. 3b and blue and green colours in Fig. 3a).

Returning to Table 2, the total cork oak forest cover loss of 8% is the result of 18% observed loss and 10% of observed gains. However, what proportion of the changes found by Dixon’s index are significant?

We found that 73% of the observed losses were significantly different to those predicted by random labelling (Monte Carlo p-value of the *C* statistic < 0.05), losses equivalent to 1369 ha; while 39% of the observed cork oak forest cover gains are significantly different to those predicted by random labelling (Monte Carlo p-value of the C statistic < 0.05), equivalent to 445 ha. Consequently, we found 924 ha of statistically significant losses which is a larger value than the 810 hectares (8%) reported in Table 2. This is due to the low significance of most of the cork oak forest gains, which does not mean that they are labelling errors, but that these areas are isolated in space and time and therefore do not show patterns of segregation (instead they show a high level of sparsity). This is also confirmed by the results shown in Table 3, where for positive *Saa* the individual cover-area statistic (*z*_*aa*_) confidence interval contains both positive and negative values. Interestingly, for both *z*_*aa*_ and *Saa* the intensity of change is similar when considering the absolute values for the median of the two statistics. This is probably due to the nature of the collection of data, which is done per area instead of per individual point (reducing mixture of points).

**Table 3:** Global median and 95% confidence interval (CI) for Dixon’s indices for areas with negative and positive *Saa*.

	Median *Saa*	CI *Saa*		Median *z*_*aa*_	CI *z*_*aa*_	
Negative *Saa*	−0.81	−2.97	−0.04	−1.13	−3.76	−0.41
Positive *Saa*	1.08	0.05	2.86	−1.15	−4.93	0.14

### Spatially explicit binomial-logit generalised linear model

**Figure 4:**
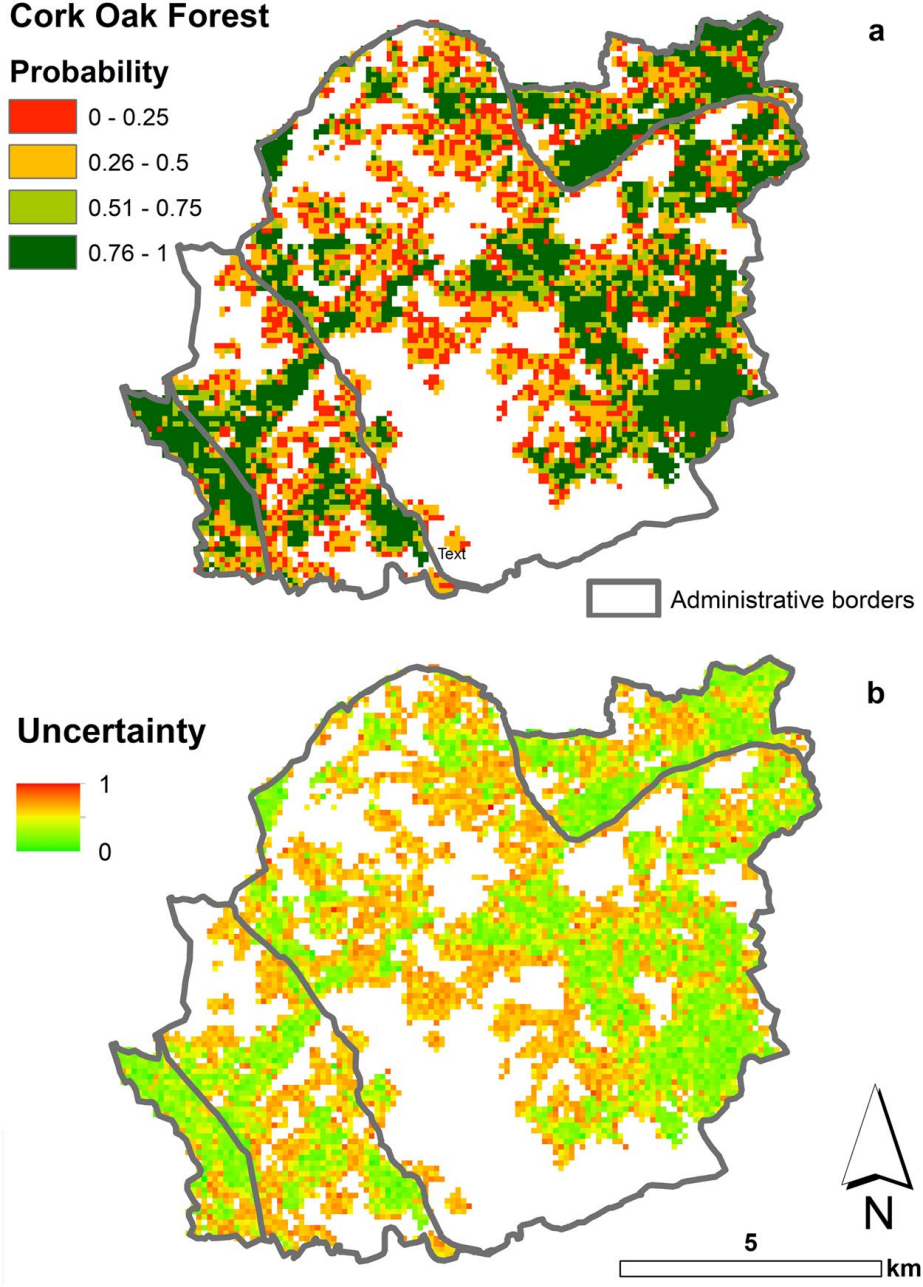
Cork oak forest cover probability of presence in 2016. Panel a: probability of presence of cork oak forest cover as obtained from the spatially explicit binomial-logit generalised linear model. Panel b: standardised uncertainty where 1 means largest interquartile range of the probability at that location. Maps were made using ArcMap 10.4 (http://desktop.arcgis.com/en/arcmap/). Source administrative limits: http://dati.regione.sardegna.it/dataset/limiti-amministrativi-comunali under CC-BY-4.0 licence.

The map produced by the spatial binomial-logit generalized linear model (Fig. 4a) shows the probability of the presence of cork oak forest in 1998 and 2016. This map confirms the same geographic change patterns obtained from the Dixon’s index of segregation, with low probability in those areas where cork oak forest disappeared, and high probability where cork oak forest was stable (no change between 1998 and 2016). Comparison between the two methods shows two differences: (i) with the binomial-logit model it is possible to state the probability that cork oak forest cover is present in an arbitrary location but not its rate of segregation or association (which is provided by Dixon’s index map); (ii) areas with decreased or increased cork oak forest are both characterised by low probability of presence (because cork oak forest was present only once during the two surveys). In fact, the uncertainty map (Fig. 4b) shows medium and high uncertainty for any area experiencing a change in cork oak forest cover. Overall, the spatially explicit binomial-logit generalised linear model is confirmed as a method that efficiently maps changes or presence of a class, and whose accuracy increases with larger numbers of available surveys over a given time period^25,54–56^.

### A geostatistical additive model for Dixon’s index segregation values

A linear geostatistical model was fitted to the significant *Saa* values using a Matern spatial covariance function with kappa (modified Bessel function of the second kind) equal to 1.5 and estimated range of 1691m (see Supplementary Fig. S1). Table 4 shows the results for this model. Almost all the parameters estimated by the model are significant, i.e. the coefficients of the covariates and spatial correlation are significantly different from zero when considering the 95% confidence interval. Only the coefficient for agriculture is not significant. This result is expected since changes from cork oak to agriculture are smaller than 0.01%. The sign of the variable’s coefficients indicates an inverse relationship between natural pastures and other forest covers and cork oak forest cover, with more segregation (cork oak forest cover erosion) in areas with increased natural pastures or other forest. The most intense changes were from cork oak forest to other forest when looking at the absolute value of the estimated parameter.

**Table 4:** Parameter estimates, standard errors and confidence intervals obtained from the linear geostatistical model of significant *Saa* values.

	Estimated parameter	Standard error	95% Confidence intervals
Intercept	0.734	0.008	0.727 0.758
Agricultural	0.001	0.011	−0.002 0.024
Other forest	−2.197	0.018	−2.232 −2.161
Natural pastures	−1.409	0.021	−1.450 −1.368
Nugget (τ^2^)	0.151		0.148 0.155
Spatial variance	1.262		1.075 1.481
Spatial range (m)	1691		1424 2009

## Discussion

This work developed a new version of Dixon’s index of segregation that accounts for the temporal dimension, edge correction and spatial heterogeneity. Previous works focused on developing the index for species interactions studies^18,37^ and for different geometric configuration of the points in space^44,57^, but not for a time dimension or for local estimations of the index. However, applying Dixon’s index to time opens up a multitude of study areas for its possible application; from land cover/use change to clustering analysis (which may include the field of epidemiology^58^), to species interaction and distribution analyses^16^. As a local statistic Dixon’s index of segregation raises some challenges^59^ (see Limitations and Conclusions section), especially in the choice of the neighborhood size *d*, but it does allow for detailed mapping of the rates of change and their uncertainty, and therefore identifies areas experiencing statistically significant land cover erosion or intensification. The measure of uncertainty marks an important development over traditional land cover change studies that use deterministic or non-spatial models^60^. In fact, a globally significant segregation index may not be significant everywhere. We have shown that large parts of Sa Serra are affected by medium and large uncertainty in land cover change, especially on the borders between different land cover types (Figs 3c and 4b), so that drawing conclusions is not possible.

The comparison of Dixon’s index of segregation with a spatially-explicit binomial-logit generalised linear model has shown the limitation of inferential models when only two land cover surveys are available for the same area, therefore returning high uncertainty for areas where changes occurred^61^.

Change analysis by Dixon’s index confirms that there is a significant decrease in cork oak forest cover in the studied area in the last 18 years. These changes are in addition to the ones already experienced in the same area between 1954 and 1998, when 29% of cork oak forests were transformed into ‘other forest’ and ‘natural pastures’^2^. Cork oak forest erosion is a large-scale process taking place throughout its entire geographical range. Numerous studies have analysed the causes of cork oak forest cover reduction, especially in the Iberian peninsula (the area with largest surface of cork oak forests in the world), and found that the reduction was due to overgrazing, wildfires, mechanical removal of the original vegetation thus making conditions for seedling establishment unfavourable, climate change and, finally, oak decline^7–9,14,62^, often acting simultaneously^1^.

The failure in cork oak regeneration is also linked to the ecological properties of the species. In many Mediterranean areas cork oak regeneration is limited by the hot and dry summers or by the low winter temperatures of Mediterranean highlands^63–65^. In Sa Serra, the optimal natural vegetation types (ecologically) are a mixture of *Quercus ilex* (holm oak) and *Q. pubescens* (downy oak) or monospecific *Q. ilex* forests^66^. Here the cork oak coexists with holm oak, downy oak and Mediterranean shrubs. However, the regenerative capacity of holm oak in these ecosystems is superior to the one of cork oak and downy oak trees^63^. This means that, in Sa Serra and in the absence of interventions that can affect the tree’s regeneration, it is expected that the vegetation will evolve in monospecific holm oak forests. The latterly described vegetation dynamics are not unique to Sa Serra or Sardinian woodlands^67^. For example, in Tuscany, Branco and Ramos^1^ reported that “*in absence of coppicing, vegetation tends toward a holm oak–dominated forest in which cork oak is shaded out. In managed situations, cork oak is the dominant tree of a lower, open maquis, with heliophylous shrubs including myrtle (Myrtus communis), broom (Cytisus villosus), lavender (Lavandula stoechas), and rockrose (Cistus spp.)”*. Similar findings are described for the cork oak forests in Sicily^68^, which with Tuscany are the two Italian regions with the largest cork oak woodland surfaces after Sardinia. It is therefore clear that in certain Mediterranean areas the continued presence of cork oak forests is only possible if they are protected, i.e. if the regeneration is supported by human interventions such as the removal of other trees and controlled fires, in order to reduce land competition from shrubs and other tree species. Controlled fires do not jeopardize cork oak presence, due to the fire-resistant properties of the cork bark and the ability of the cork oak trees to produce fast-growing roots and basal suckers^1,69^. These interventions are routinely applied in those cork oak forests that are exploited by the cork industry^10,70^. In fact, clean areas around cork oak trees allow for easier extraction of the cork bark and the integration of livestock or agricultural activities within the cork oak forest system (an agro-forestry system, or Dehasas in the Iberian Peninsula)^71^.

In Sa Serra, as in other regions (e.g. in Portugal^7^), a potential reason for the transformation of cork oak forest cover to ‘other forest’ is that the cessation of human intervention has led to an absence of cork oak regeneration. This is mainly the result of population relocation from rural to urban areas with an associated reduction in agricultural activities, including cork extraction^26^ (Table 2). Urbanization with movement of people from countryside to cities is a phenomenon usual in non-coastal areas of Sardinia^72,73^. It is, therefore, a consequence that land abandonment facilitates the growth of Mediterranean shrubs and other trees such as holm and downy oaks, with the ensuing natural conversion of monospecific cork oak forest to mixed forests^74^. As described in the Methods, the ‘other forest’ class can contain up to 25% of cork oak tree canopy cover; this means that a change in this definition that thus reduces the minimum amount of cork oak canopy cover required to be classed as ‘cork oak forest’, may also reduce the number of areas where cork oak forest changed to ‘other’ forest. Although a cork oak canopy cover of 10% is still sufficient for the definition of individual cork oak forest by the FAO-FRA international forest classification, the limit of 25% maintains consistency with the classification of cork oak forest adopted by the Sardinian Regional Land Use Census^75^, and allows the integration of our findings with the current official Sardinian land use products.

Land abandonment has not only favoured the conversion of cork oak forest to other forest, but also the expansion of natural pastures in areas previously occupied by agricultural and cork activities (e.g. the center of Sa Serra, Figs 2 and 3). While historically the industrial production of wine bottle stoppers from cork promoted the diffusion of traditional agro-forestry^67^ based on cork oak trees, the development and intensification of new cheese-making techniques in the first decades of the 20^th^ century (e.g. to produce Pecorino Romano, a mature cheese made from sheep’s milk that is exported mainly in Europe and US^76^) has increased the numbers of livestock and hence the pressure on cork oak forest systems^77–79^. In the last 100 years, the number of sheep increased from two million to three million units^80,81^. In Sa Serra over the same period, the number of sheep increased by 20%, from 98000 in 1929 to 118000 units in 2010. The economical profitability of the sheep milk, the European Commission (EU Regulation n. 1030/2009; EU Regulation n. 1144/2014) and Sardinian Regional Government (Sardinian Rural Development Plans 2007–2013 and 2014–2020) agricultural policies in support of milk farming, and the decreasing cork prices, pushed many farmers to convert their activities to dairy sheep, with subsequent replacement of agricultural and agro-forestry lands into high density evergreen stands^67^. Today, Sardinian territories contain 40% of all Italian sheep-milk livestock^80^ while Sardinian cork production halved from 17000–20000 tons in the years 1960–1970 to just 8000–10000 tons in the decade 2000–2010^82,83^, further indicating an abandonment of cork oak forest systems.

In many cork producing countries, as in Sardinia, cork oak woodlands of a certain density are legally protected by national and regional legislations since 1956 (National Law n. 759/1956; Regional Law n. 4/1994; Regional Law n. 8/2016, accessible from http://www.normattiva.it/). These laws regulate the pruning of cork oak trees and the extraction of cork barks, and commit the national and regional governments to the adoption of forest management plans and reforestation activities. However, these laws do not regulate the grazing loads in cork oak forests, and require the adoption of compulsory management plans only for public forests, not for private farms. The Habitats Directive (European Economic Community Directive 92/43/EEC) also aimed to protect cork oak woodlands (Habitat 9330, *Q. suber* forest) and their agroforestry systems (Habitat 6310, Dehesas) due to their importance in biodiversity conservation^84^ but no part of Sa Serra has ever been proposed for inclusion in the Natura 2000 network (http://www.minambiente.it/pagina/cartografie-rete-natura-2000-e-aree-protette-progetto-natura) as a Site of Community Importance (SCI). However, even in SCI regions cork oak tree presence is not protected. A recent study in Sardinia showed that in the presence of grazing livestock, cork oak forest inside and outside SCI areas are affected by lower regenerative capacity^85^, with estimated extinction of cork oak-based habitats in 100–125 years. This is because most of the SCI areas exist on the official cartography only and the accompanied SCI management plans do not contain severe restriction to protect the forests (e.g. grazing load, sustainable agriculture interventions, subsides for forest conservation). In addition, farmers are often unaware of whether their land is within a SCI area or not^85^.

In conclusion, cork oak forest regeneration in Sardinia relies on human intervention for the protection of cork oak seedlings from intensive grazing and other vegetation competition. The option of doing nothing, e.g. “leave the forest as it is” is not likely to work. Cultural and political actions are much needed to preserve this historical component of Sardinian landscapes.

## Limitations and Conclusion

The Dixon’s index of segregation was applied to land cover changes and extended to the temporal domain; a pioneering use of the index that allowed direct mapping of changes and interpretation of the intensity of these changes. In fact, in the seminal work of Dixon^18^ and its further development by Ceyhan^57^ the temporal dimension, the inclusion of which determines a change in the point number of points sharing the same neighbourhood, was not considered. Our new work also provided a measure of uncertainty at each point location by calculating the relative significance of the segregation values^86^. All these properties together make the Dixon’s index of segregation superior to common methods such as confusion matrices or simple intersection of two or more images^23,24^, or those that do not provide confidence levels or measures of uncertainty. While it is the only option in the absence of covariates, geostatistical model-based approaches should be preferred in the presence of explanatory variables^87^.

One of the main limitations of the use of the local Dixon’s index of segregation (when compared to inferential models) is the choice of the neighbourhood window, *d*, which requires several simulations to optimize it. In addition, this optimization requires that candidate values for *d* are sufficiently different (e.g. 250 m and 260 m may result in the same accuracy for the index)^88^. Another limitation is the computational time (as with most spatially explicit methods). Sa Serra is a relatively small area (about 100 km^2^), and the algorithm took about 4 hours to run through about 40000 points (i.e. 10000 points per hour, where for each point the neighbourhood of 250 m contained 100 points). This may require long computation times for larger areas (regions or countries), however, parallelization is the ideal solution since the results of each neighbourhood are independent from the others^89^. Finally, Dixon’s index of segregation cannot account for multitemporal changes (i.e. comparing more than two time slots) or for explanatory variables, i.e. environmental forces, for which spatially explicit regression-like approaches are the ideal alternative, although results have a different interpretation (Dixon’s index of segregation provides a direct interpretation of the clustering nature of the change process)^90^.

A non-methodological but applied limitation is that this analysis only considered changes between broad-defined land cover classes. While this may prevent inaccuracies in the classification and area delineation, in the other hand it may not represent the real nominal changes happening in the area^91^.

This work found a slower but active erosion and degradation of the Sa Serra cork oak forest than that of the 1954–1998 period, though European and Italian forest cover is actually increasing^12^. The Sardinian law on cork oak woodland protection passed in 1994 (http://www.regione.sardegna.it/j/v/86?v=9&c=72&file=1994004) prohibits the destruction of cork oak forests but allows, once authorized, felling of single trees. This law has certainly contributed to the conservation of cork oak forests in Sardinia by slowing down their destruction/erosion (when compared to the 29% cork oak forest losses from 1954 to 1998), however, removal of single trees, often at the borders of the forests, has resulted in the erosion of coverings at the edge of the cork oak forest^92^.

The future of cork oak forests may be a different story. The very recent policies concerning the diversification of agricultural and forestry products in order to obtain sustainable systems linked to agricultural and non-agricultural sources of income (cork, meat, milk, wool, rural tourism and education in farms), ecosystem services such as biodiversity hotspots, control of desertification, soil conservation and carbon sinking capacity^93^, are an opportunity for stopping the current regional trends in cork oak forest cover destruction^92,94,95^ and recovering cork oak forests and agro-forestry systems that otherwise are economically not profitable in short term^1^.

## Electronic supplementary material

Supplementary Figure S1

Dataset 1

## Data Availability

All data generated or analysed during this study are included in this published article and its Supplementary Information files (Supplementary Dataset 1).
