# Supplementary material for: Employing a spatio-temporal contingency table for the analysis of cork oak cover change in the Sa Serra region of Sardinia
Source: Sci Rep. 2018 Nov 16;8:16946. doi: 10.1038/s41598-018-35319-1 (PMC6240039; doi:10.1038/s41598-018-35319-1)
Supplement: Supplementary file 1 — Supplementary Figure S1 [file 41598_2018_35319_MOESM1_ESM.pdf]

Employing a spatio-temporal contingency table for the analysis of cork oak cover change in the Sa Serra region of Sardinia.

Sandro Dettori, Maria Rosaria Filigheddu, Giovanni Deplano, Juan Escamilla Molgora, Maddalena Ruiu & Luigi Sedda

Supplementary Information.

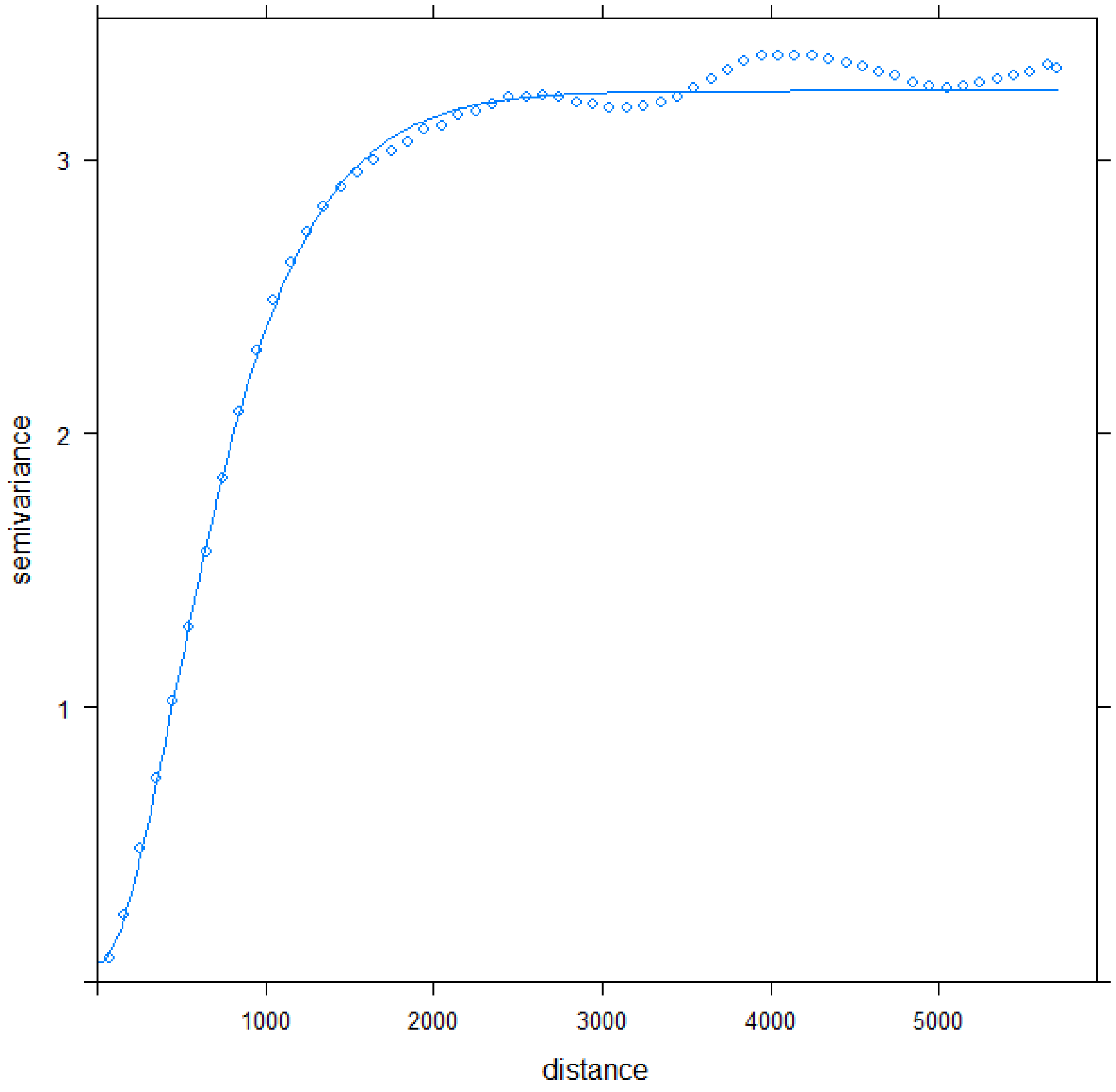

Figure S1. ***Saa* variogram**. Experimental (dotted) and fitted (line) variograms employed in the linear geostatistical model. Graph was made using R-cran 3.5.0 (http://r-project.org).
